# Current State of Applications of Nanocellulose in Flexible Energy and Electronic Devices

**DOI:** 10.3389/fchem.2020.00420

**Published:** 2020-05-21

**Authors:** Otavio Augusto Titton Dias, Samir Konar, Alcides Lopes Leão, Weimin Yang, Jimi Tjong, Mohini Sain

**Affiliations:** ^1^Centre for Biocomposites and Biomaterials Processing, University of Toronto, Toronto, ON, Canada; ^2^Department of Mechanical and Industrial Engineering, University of Toronto, Toronto, ON, Canada; ^3^College of Agricultural Sciences, São Paulo State University (Unesp), São Paulo, Brazil; ^4^College of Mechanical and Electrical Engineering, Beijing University of Chemical Technology, Beijing, China

**Keywords:** cellulose nanofibrils, cellulose nanocrystals, luminescent materials, conductive materials, films, nanocomposites, energy storage device, hybrid materials

## Abstract

Novel and unique applications of nanocellulose are largely driven by the functional attributes governed by its structural and physicochemical features including excellent mechanical properties and biocompatibility. In recent years, thousands of groundbreaking works have helped in the development of targeted functional nanocellulose for conductive, optical, luminescent materials, and other applications. The growing demand for sustainable and renewable materials has led to the rapid development of greener methods for the design and fabrication of high-performance green nanomaterials with multiple features, and consequently new challenges and opportunities. The present review article discusses historical developments, various fabrication and functionalization methods, the current stage, and the prospects of flexible energy and hybrid electronics based on nanocellulose.

## Introduction

**Figure 1:**
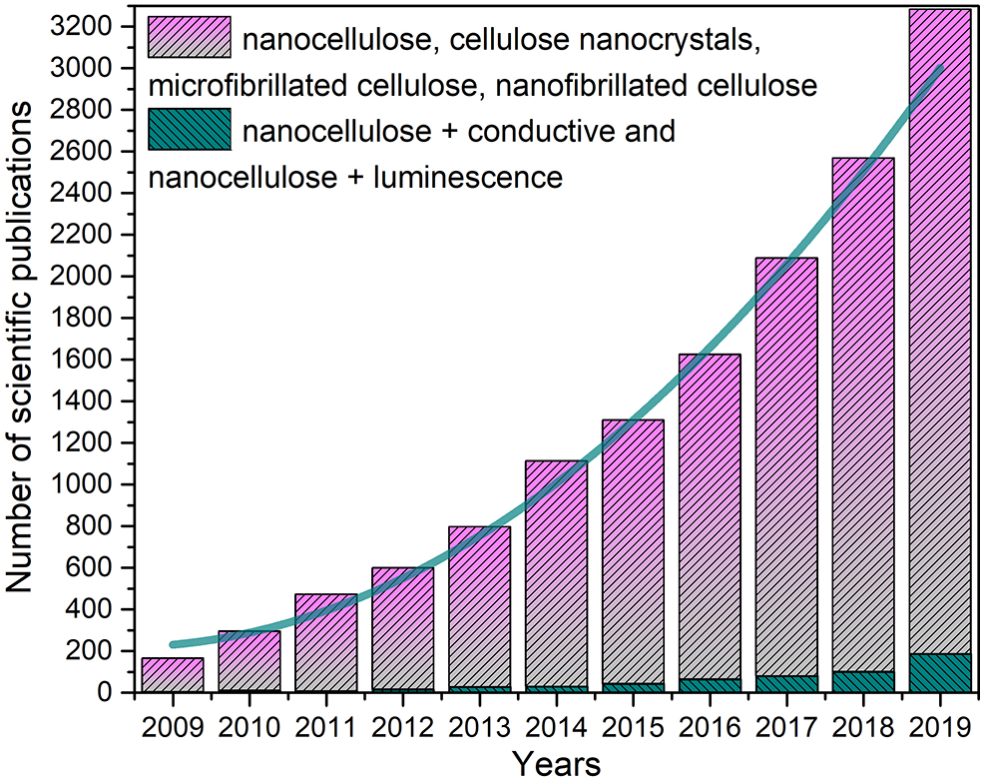
Number of publications in the area of nanocellulose. Data analysis performed on SciFinder using terms nanocellulose, cellulose nanocrystals, microfibrillated cellulose, nanofibrillated cellulose.

Nanocellulose has received significant interest due to its mechanical and optical properties, biodegradability, availability, recyclability, renewability, and low coefficient of thermal expansion. This nanomaterial, in the form of either nanofibrils or nanocrystals, has been reported as an interesting sustainable and tunable platform for the production of a variety of high value products (Kumari et al., 2014). As shown in Figure 1, there is an exponential trend in the number of citations in the area of nanocellulose. Considering the aforementioned properties of nanocellulose, transforming this renewable material into advanced materials is a crucial step toward sustainable development (Giese et al., 2014). This is feasible due to the presence of a large number of hydroxyl groups within the nanocellulose structure as well as its high surface area, high aspect ratio and great axial modulus, making this fascinating biopolymer a more attractive substrate than traditional cellulose (Wang et al., 2014; Rusmirović et al., 2017). All forms of nanocellulose are recognized as being more effective than macroscale fibers in terms of reinforcement effect and reactivity (Azeredo et al., 2010). The benefits of using renewable materials such as nanocellulose is that they can replace plastic and metal substrates in a wide range of applications and consequently diminish the pollutant residues in the environment (Huang et al., 2013). In addition, there has been rising concern over dependence on finite and depleting non-renewable petroleum and metal resources. In this sense, the design and development of green technology from advanced hybrid nanomaterials have become a key principle of ecological sustainability (Khalil et al., 2012). Currently, there is a high demand for flexible, transparent, strong, sustainable and functional materials (Österberg et al., 2013; Abdul Khalil et al., 2014). However, despite various advantages, nanocellulose exhibits limitations that restrict its widespread applications, such as poor thermal stability, incompatibility with hydrophobic polymers and absorption of moisture. In order to employ nanocellulose for high value materials, the current challenge is providing additional functionalities via covalent modification, blending, cross-linking, polymerization, or even a combination of these methods. In this context, the presence of a large number of hydroxyl groups within the nanofiber's structure may act as a structured platform for surface modification through different substituents (Habibi, 2014). The combination of nanocellulose and carbon-based materials such as graphene, carbon nanotube and carbon dots have been advocated as a promising approach for the future of renewable electronics. In this review, we discuss the most recent developments of flexible energy and electronic devices, with special focus on luminescent and conductive materials based on nanocellulose, and potential design strategies required to expand its use in commercial applications.

### A Basic Introduction of Nanocellulose

#### Brief Description of Nanocellulose Variety and Nanocellulose-Based Materials

**Figure 2:**
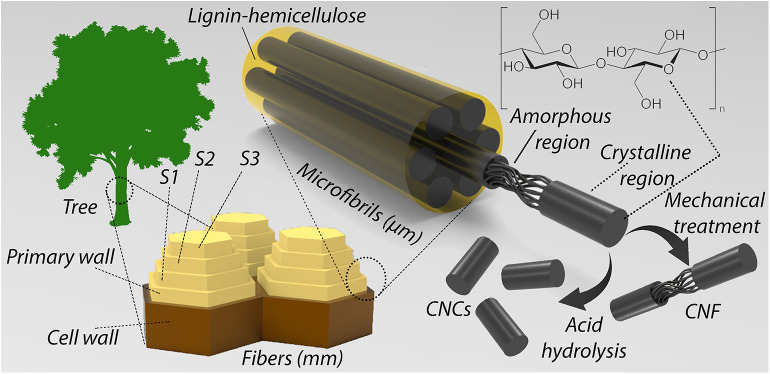
Hierarchical structure of cellulose and schematic illustration of crystalline and amorphous regions in cellulose fiber.

Cellulose is the most abundant natural polymer on Earth (Bilodeau et al., 2014). The cell wall of a fiber is built from external primary (P) and inner secondary walls (S1, S2, and S3) (Kargarzadeh et al., 2017). As shown in Figure 2, cellulose fibers can be defibrillated and/or hydrolyzed into smaller nanoscale entities known as nanocellulose, which occurs in the form of cellulose nanocrystals (CNCs) or cellulose nanowhiskers (CNWs), and nanofibrillated celluloses (NFCs) or cellulose nanofibers (CNFs) as they are alternatively named.

**Figure 3:**
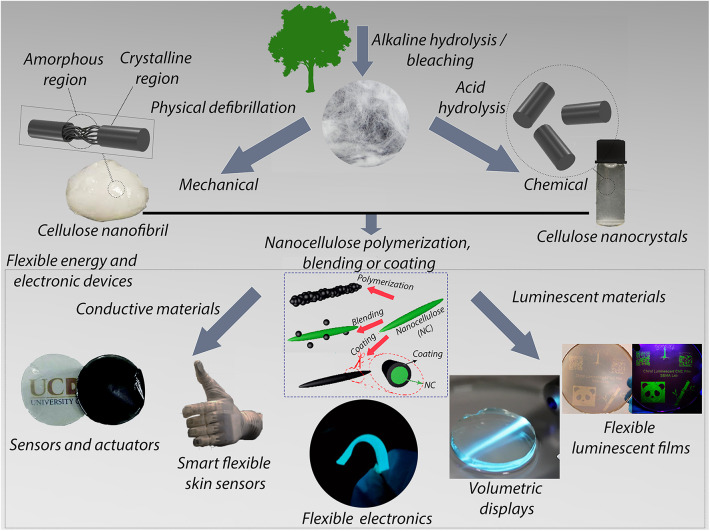
Scheme of CNC and CNF extraction from cellulose, surface functionalization of nanocellulose for application in flexible energy and electronic materials.

Nanocellulose has been employed either in pristine form or through modification of its surface. CNFs and CNCs have distinct properties in terms of aspect ratio, surface area, and structure. CNFs possess the amorphous domains facilitating the cellulose chains motion which consequently lead to mechanical robustness and flexibility making it suitable for free-standing thin films manufacturing. On the other hand, CNCs can form a percolation network that provides a conduction path and reinforcing effects enhancing energy transfer, because of their higher crystallinity, aspect ratio and specific surface area. In addition, CNCs can act as multifunctional nanoplatforms for emerging biomedical applications and light emitting devices. The surface modification of cellulose nanoparticles is typically achieved by physical interactions or through covalently bonded of molecules with nanocellulose. CNFs in their pristine form are typically employed in hydrogels for medical applications and as reinforcing agents in hydrophilic polymers for food packaging. Hydrogels based on cellulose derivatives have numerous advantages due to their intrinsic biocompatibility and excellent gel-forming properties (Ahmed and Ikram, 2016). For hydrogels, mechanical property and cell type are crucial features for practical applications in such fields as tissue engineering (Borges et al., 2011; Martínez Ávila et al., 2014), drug delivery (Valo et al., 2013), contact lenses (Tummala, 2016), and water treatment (Gatenholm and Klemm, 2010; Lin et al., 2015). On the other hand, CNCs are rarely employed without surface modification since the absence of amorphous phase make the resulting CNC film brittle limiting the scope of the application to incorporation of CNC into hydrophilic polymers such as PVA (Jahan et al., 2018), PEO (Surov et al., 2018), and epoxy resins (Girouard et al., 2015). Due to the hydrophilic nature of nanocellulose, it can absorb water under environment conditions and may deteriorate the mechanical properties, and also impair its compatibility with hydrophobic polymers (Wang and Drzal, 2012; Farahbakhsh et al., 2015). This is evidently the biggest limitation that restricts nanocellulose utilization for advanced materials. In this context, numerous studies have been focused on the development of greener strategies for the synthesis of functional nanomaterials based on nanocellulose. As shown in Figure 3, both CNF and CNC have been modulated by different strategies in order to add new functionalities to the nanomaterials and consequently broaden their applicability. The combination of multiple components with specific features and their integration into a single device appears to be a promising approach for next-generation flexible electronics.

## Surface Modification OF Nanocellulose

The large specific surface area and the presence of a large number of hydroxyl groups within the nanocellulose structure make these nanofibers an exceptional platform for surface modification through various chemistries (Habibi, 2014). The above-mentioned features of nanocellulose offer attractive strategies for targeted surface modification to incorporate almost any desired surface functionality. During the 1920s and early 1930s, the hydrophobization of cellulose surface was achieved via esterification reactions, and used for food packaging applications (Hofmann and Reid, 1929). Over the last decades, several strategies have been employed to achieve hydrophobicity via acetylation and esterification of chitin (Nair et al., 2003), starch (Jeon et al., 1999), xylan (Peng et al., 2010), cellulose nanocrystals (Yuan et al., 2006), cellulose fibrils (Huang et al., 2012), and cellulose fibers (Vaca-Garcia et al., 1998). The hydrophobization of nanocellulose has been extensively explored, but studies toward more sophisticated functionalization for advanced applications have attracted more and more attention and the research is still in its early days. A variety of long-chain aliphatic compounds have been grafted on CNFs and CNCs via different techniques in order to reduce moisture absorption (Sethi et al., 2018), enhance interfacial affinity between nanofibers and resins (Tan et al., 2015; Yoo and Youngblood, 2016; Trinh and Mekonnen, 2018), and improve thermal stability (Sharma and Deng, 2016). Over the years, more sophisticated products based on cellulose derivatives have started to be considered as part of next generation technology. Recently, breakthrough products based on nanocellulose such as artificial skin, biosensors, stretchable circuits, and volumetric displays have been extensively investigated. To achieve this goal, it is necessary to modify cellulose structure and give it new characteristics. The concept of creating ion exchangeable carboxyl groups via TEMPO-mediated oxidation on nanocellulose surface have been employed by several authors (Cheng et al., 2016; Wei et al., 2016; Raghuwanshi and Garnier, 2019; Tao et al., 2020; Xu H. et al., 2020; Zeng et al., 2020; Zhang et al., 2020a,b; Zhu et al., 2020). TEMPO-oxidized nanocellulose (TONC) is recognized as a potential platform for emulsifiers (Goi et al., 2019), carbon dot anchoring (Jiang et al., 2016), fluorescent sensors (Wang et al., 2020b), conductive devices (Zhang et al., 2020b), and contributing to lower thermal expansion of materials (Fukui et al., 2018). The preparation of TONCs has therefore opened up the possibility of producing novel functional materials (Yang et al., 2018). Alternatively, nanocellulose has been modified by grafting technique either via one- (Vadakkekara et al., 2020) or two steps methods (Anirudhan and Rejeena, 2014; Mandal and Chakrabarty, 2015). Graft polymerization of vinyl monomers on cellulose structures, for example, can provide active sites for the attachment of desired functional groups (Kumari et al., 2014; Tong et al., 2020). Chemical and physical cross-linking have been employed for improvement of certain characteristics such as moisture sensitivity of nanocellulose-based materials (Sharma and Deng, 2016) and for the fabrication of fascinating materials such as flexible aerogels (Wu et al., 2019; Shahzamani et al., 2020). Functional coating on the nanofiber surfaces has also been investigated by evaluating the adhesion between nanofiber and a variety of nanoparticles (Wang Z. et al., 2019). Core–shell tannic acid and dopamide coatings on CNFs are among the recent methods for nanocellulose functionalization (Nguyen et al., 2016; Wang et al., 2017).

## Nanocellulose Derived Conductive Materials

Conductive materials predominantly contain metal particles, carbon materials, and conductive polymers. Nanocellulose can be combined with these conductive materials to fabricate conductive composites with high mechanical strength, stiffness, foldability and flexibility (Pottathara et al., 2016). The fabrication of nanocellulose-based conductive composite has been achieved mostly by surface grafting and blending of conductive species and nanocellulose (Ko et al., 2017). The surface grafting method is widely employed to covalently incorporate conductive materials onto nanocellulose as biotemplate. On the other hand, blending method is used to mix different materials in which the strength and charge-transfer of individual materials will be controlled by the physical interaction between blend components. Currently, conductive carbon nanomaterials and metallic nanoparticles are often blended with nanocellulose to produce conductive hybrid materials (Du et al., 2017). However, more sophisticated techniques have been developed to fabricate nanocellulose-based conducting materials.

### Combination of Nanocellulose and Conductive Polymers

Nanocellulose is a promising sustainable material for electronics in combination with conducting polymers (Zhang et al., 2013). Green and biodegradable flexible conducting polymers have been attracting attention for potential applications in electrodes and display devices (Shi et al., 2013). Self-healing, flexible and biocompatible electroconductive hydrogel based on nanocellulose has been fabricated to be employed in implantable electronic devices (Han et al., 2019a). The poor solubility of conjugated monomers such pyrrole and aniline in organic solvents typically leads to aggregate formation and consequently brittle materials (Sakakibara and Rosenau, 2012). In this context, the combination of the ecofriendly attributes, strength, and flexibility of the nanocellulose, along with the conductivity and thermal stability of the conducting polymers has opened opportunities to develop a new class of advanced materials toward a more sustainable future (Hai and Sugimoto, 2018; Dias et al., 2019). Combination of conjugated conducting polymers and cellulose was reported for the first time by Bjorklund and Lundström (1984). In the early days of conducting polymer/ polysaccharides composite research, limited functions were assigned to the fabricated nanocomposite being often used for sensing applications (Kelly et al., 2007). In the early 2010s, chitin was blended with polyaniline (PANI) for humidity sensing applications (Ramaprasad and Rao, 2010). A novel technique for *in-situ* polymerization of aniline onto chitin was reported by Marcasuzaa et al. (2010) soon afterwards. Recently, chitin and chitosan were used as a biotemplate to fabricate conductive materials based on thiophene (Hai and Sugimoto, 2018). Conductive and luminescent elements were grafted on cellulose via two-step oxidative graft copolymerization of fluorene and thiophene (Phung Hai and Sugimoto, 2017). The control of luminescence and conductivity was achieved varying the fluorene/thiophene ratio. Bhowmik et al. (2016) proposed a vapor deposition polymerization of aniline on cellulose paper via oxidation process. In recent decades, studies toward the fabrication of more robust conductive biomaterials with high mechanical strength, increased flexibility and foldability have been conducted to produce high-performance flexible materials. This contributed to the rapid expansion of the use of nanocellulose as a sustainable substrate for advanced applications in the electronics industry. Recently, a homogeneous coating of polypyrrole (PPy) on bacterial cellulose nanofibers was successfully achieved by *in-situ* chemical polymerization (Lay et al., 2017a). The obtained nanofilms displayed a high conductivity of 1.22 S.cm^−1^ and tensile strength of about 162 MPa. Hafez et al. (2017) and Ko et al. (2017) utilized an environmentally friendly approach to produce nanocellulose-based conductive materials in aqueous solution. Poly(3,4-ethylenedioxythiophene)/poly(styrene sulfonate) (PEDOT:PSS) acted as conductive layer on the cellulose nanofibers. In another study carried out by Lay et al. (2017b), flexible, lightweight, and strong conductive nanofilms were prepared by cellulose nanofibril (CNF) and PEDOT:PSS mixing followed by *in-situ* oxidative chemical polymerization of pyrrole. The synergistic effect between PEDOT:PSS and PPy offered high electrical conductivity (~11 S.cm^−1^) and specific capacitance (~315 F.g^−1^) to the ternary hybrid material. Recent work demonstrated that it is possible graft thiophene by *in-situ* polymerization onto as-prepare CNF films. According to the authors, the insulating nature of the produced nanocellulose films changed to semiconductor after oxidative polymerization (Dias et al., 2019).

**Figure 4:**
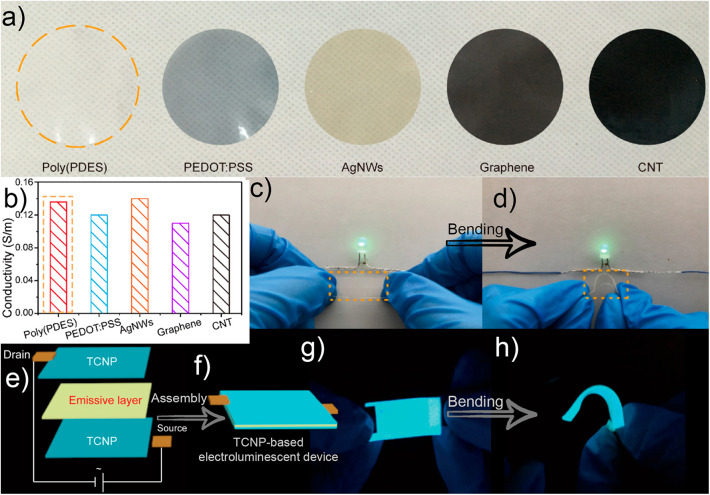
**(a)** optical photograph and conductivity **(b)** of nanofilms prepared from CNF with poly(PDES), and other conductive elements; digital photographs of horizontal **(c)** and curved **(d)** CNF/poly(PDES) film with the lighting LED; and **(f)** Schematic illustration of CNF/poly(PDES) flexible electroluminescent devices; and photographs of the flexible CNF/poly(PDES) electroluminescent devices with **(g)** horizontal, and **(h)** curved configuration. Adapted with permission from Zhang et al. (2020a). Copyright (2020) American Chemical Society.

A well-defined core-shell structured PANI/CNF produced strong synergistic interactions with soy protein isolate via tannic acid crosslinking. The interconnected network strategy offered improved electrical conductivity (0.078 S.m^−1^), foldability and enhanced structural stability, which make these nanofilms attractive for flexible and green energy-storage systems (Wang Z. et al., 2019). Shimizu et al. (2019) found that the thermal and electrical properties of TONFC films were optimized using a counter-ion exchange process. The thermal conductivity of the TOCNF films increased in the case of the shorter alkyl chain of the quaternary alkyl ammonium. Fu et al. (2020) prepared highly conductive films via pyrrole *in-situ* polymerization on CNF films by soak and polymerization method. The flexible film exhibited high electrical conductivity of about 24 S cm^−1^, high tensile strength up to 72 MPa, and superior thermal stability. In addition, the hybrid nanofilm as a supercapacitor electrode featured a high capacitance and a capacitance retention of 70.5% after 5,000 cycles. Unuma et al. (2019) investigated the conductivity variation of flexible CNF/ PEDOT: PSS composites in the THz region and how the conductivity can be controlled. The resulting nanofilms exhibited larger carrier densities as the PEDOT: PSS content increased. The classic trade-off between the transparency and conductivity encountered in nanofilms incorporated with carbon materials and conjugated polymers was addressed by Zhang et al. (2020a). The authors were able to fabricate a highly transparent nanofilm (transmittance of about 95%) with conductivity comparable to those of graphene and PSS: PEDOT. More specifically, TEMPO-mediated oxidation of CNF followed by *in-situ* photopolymerization of polymerizable deep eutectics solvent (PDES) monomer on its surface were employed in the fabrication of the nanofilm and resulted in exceptional mechanical and electrical durability. The authors revealed negligible effect on the resistance of the transparent nanofilm after 6,000 bending cycles at a 150° bending angle. In addition, the authors fabricated a robust device assembling the nanofilm into a flexible electroluminescent device as shown in Figure 4.

### Combination of Nanocellulose and Conductive Carbon Materials

**Figure 5:**
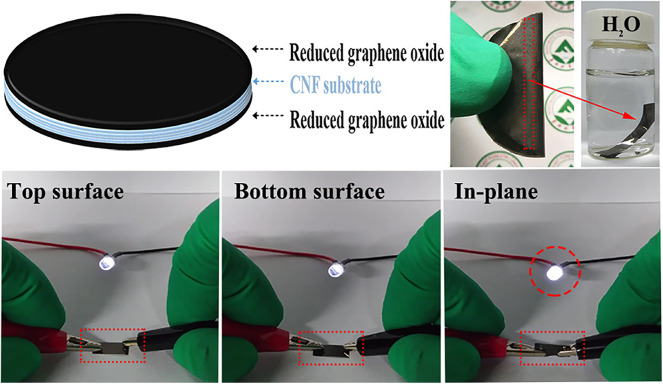
Schematic of the sandwich configuration of the RGO/CNF/RGO nanofilm and LED lamp demonstration in different regions of the film. Reprinted with permission from Hou et al. (2018). Copyright (2018) American Chemical Society.

Novel carbon materials such as those made of graphene and carbon nanotube (CNT) have received wide attention due to their high surface area, excellent electrical conductivity and ultralight weight (Zheng et al., 2015; Graziano et al., 2020). The combination of nanocellulose and carbon nanoparticles can improve the mechanical and conductive properties of the composite. The simple blend of nanostructured carbon materials and nanocellulose has been conducted over past years. However, the hydrophilic nature of nanocellulose and its marginal affinity with graphene, for example, make the blend method impractical for commercial applications. In this sense, layer-by-layer (LbL) method for the self-assembly of functional materials onto cellulose materials has been accomplished (Hamedi et al., 2013). This technique has the potential to produce thin-film devices with a variety of functions such as gas and chemical sensing and volumetric light emission. Nyström et al. (2015) proposed the construction of a 3D crosslinked nanocellulose-based supercapacitor with carbon nanotube electrodes using a self-assembly method. The resulting aerogels were soft and highly resilient to compression. As an alternative to layer-by-layer assembly technique, a sandwich-structured conductive film (Figure 5) prepared via step-by-step vacuum filtration process exhibited the optimal design in terms of electrical and mechanical performance among the CNF/RGO (cellulose nanofibril/reduced graphene oxide) composites (Hou et al., 2018). The sandwich layers connected in parallel exhibited extraordinary in-plane electrical conductivity of about 4,400 S.m^−1^ with only 5% RGO content.

**Figure 6:**
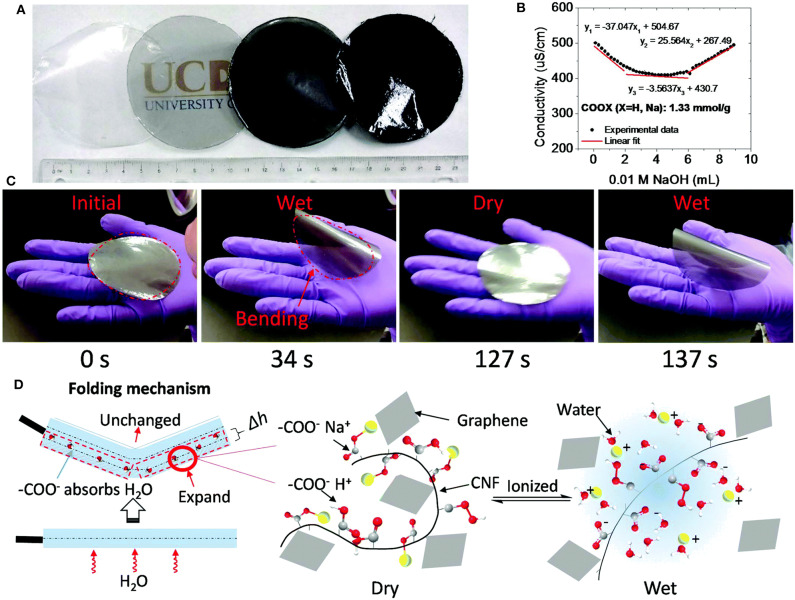
Graphene/CNF film and application in soft robots; **(A)** image of graphene/CNF films; **(B)** conductivity of carboxylated charged CNFs; **(C)** cyclic bending and recovery after exposure to human breaths at times **(D)** Expansion of CNF chains upon the charged surface groups on CNFs become ionized by water under moisture condition. Reprinted with permission from Xu and Hsieh (2019). Copyright (2019) The Royal Society of Chemistry.

As reported by Wang R. et al. (2019), conductive paper fabricated by means of ultrafiltration showed exceptional conductivity of about 120 S m^−1^ at 20% graphene oxide loading. In this study, graphene oxide and cellulose were reduced by green l-ascorbic acid prior to film formation via a one-pot method. The magnitude of conductivity of the fabricated film was similar that of traditional graphene/cellulose composites obtained using multiple steps, hazardous chemicals and very costly procedures (Wang R. et al., 2019). In an intriguing approach, amphiphilic CNF was prepared by TEMPO mediated oxidation and blending graphene with TOCNF (Xu and Hsieh, 2019). They proposed a simple, green, and inexpensive approach using amphiphilic CNF as multi-functional exfoliating and dispersing material for moisture responsive graphene nanofilm. Amphiphilic CNF performed effectively by associating with graphite through hydrophobic interactions (Figure 6). On the other hand, the hydrophilic surfaces acted as CNF-bound graphene dispersing in water and the charged carboxylates stabilized the CNF-bound graphene dispersions in aqueous solution (Xu and Hsieh, 2019). Ardyani et al. (2020) investigated the effect of incorporation of anionic and cationic surfactants in nanocomposites of reduced graphene oxide (RGO) and nanocellulose. This technique increased the electrical conductivity (1.28 × 10^−4^ S.cm^−1^) of the nanocomposites since the fractional free volume was reduced due to the enhanced affinity between RGO and nanocellulose. The cationic surfactants exhibited satisfactory ability for stabilization of RGO in CNF since the electrical conductivity of the nanocomposite improved by 143 orders of magnitude when compared to anionic surfactants. Efficient exfoliation of graphene by triethanolamine and subsequently combination with TOCNF resulted in a hybrid nanofilm with enhanced conductivity, mechanical properties, and thermal stability (Zhan et al., 2019). According to the authors, the improved performance is ascribed to the layered hierarchical structure in which TOCNFs were inserted into layers of graphene. The hexagonal-layered structure of boron nitride (BN) nanosheets has also attracted attention from academic and industrial perspectives due to their exceptional thermal conductivity and electrically insulating performance. The combination of BN nanosheets and CNFs has been proposed as flexible substrates to dissipate the heat from printed circuit boards (Hu et al., 2020). A recently discovered family of two-dimensional structure named MXenes have attracted extensive attention due to their metal-like electrical conductivity (Tian et al., 2019). In contrast to the chemically inactive and hydrophobic surface of graphene, the hydrophilic nature of MXenes may overcome the weak interaction between different components and also offers good opportunities for the surface functionalization of MXene without compromising its excellent electrical conductivity (Zhao et al., 2018). As reported by Tian et al. (2019), the large number of hydroxyl group of CNFs favored good interfacial interaction with MXene, and their combination offered a nanomaterial with high mechanical strength maintaining a high capacitance of about 300 F g^−1^ and a high conductivity up to 300 S cm^−1^ (Tian et al., 2019). The authors suggested that MXene/CNF hybrid films can be employed for structural energy storage.

### Hybrid Conductive Composites Based on Nanocellulose

**Figure 7:**
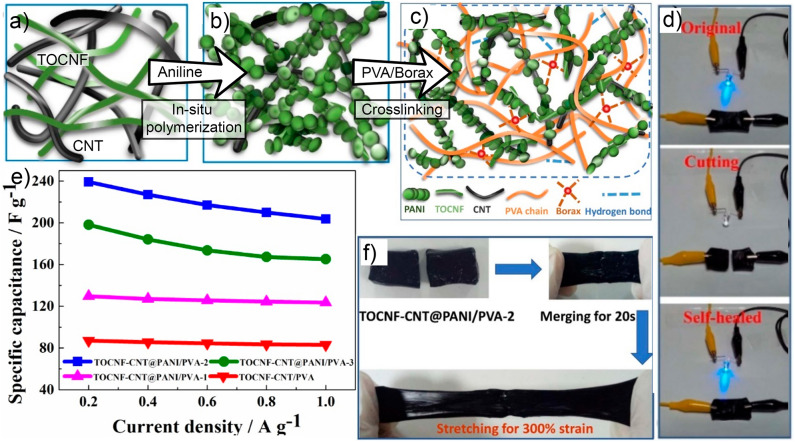
**(a)** Schematic of TOCNF/CNT composite, **(b)** aniline polymerization on TOCNF /CNT composite, **(c)** and (TOCNF/CNT)-PANI/PVA composite hydrogel, **(d)** optical images of cut and self-healed (TOCNF/CNT)-PANI/PVA hydrogels used to light up a light-emitting diode (LED) circuit, **(e)** specific capacitance of the hydrogel electrodes (at different PANI/ TOCNF-CNT ratio) under different current densities, and **(f)** illustration of self-healing property for (TOCNF/CNT)-PANI/PVA hydrogel. Adapted with permission from Wang et al. (2020a). Copyright (2020) MDPI.

Hybrid nanocomposite films made of conducting polymers, graphene and nanocellulose have been extensively studied. Recently, nanocomposites built up of two or more constituent materials have been engineered for targeted applications. Flexible layered films based on CNF, reduced graphene oxide (RGO) and polypyrrole (PPy) were prepared via a facile filtration method (Hou et al., 2019). The optimized layered configuration and the synergistic effects of three components make these hybrid films suitable for supercapacitor electrodes. The sandwich-structured film electrode showed a high specific capacitance about of 300 F g^−1^ and outstanding capacitance retention (~82% after 1,000 cycles). Conventional supercapacitors are susceptible to continued mechanical stress and deformations, and normally do not meet the design requirements of flexible wearable electronic devices (Wang et al., 2020a). Flexibility and self-healing capability have been demonstrated as important features, in addition to electrical conductivity, specific capacitance and mechanical robustness. In this context, Wang et al. (2020a) fabricated a core–shell structured TOCNF-CNT/PANI and incorporated it into self-healable PVA hydrogel matrix. As shown in Figure 7, the nanocomposite hydrogel showed excellent flexibility, electrical conductivity of about 15 S.m^−1^, and specific capacitance. The nanohybrid hydrogel electrodes exhibited great potential for development of advanced wearable electronic devices. In recent years, some efforts have been devoted to fabricate electrically conductive elastomer nanocomposites based on a natural rubber (NR) matrix. Han et al. (2019b) developed conductive elastomers based on natural rubber and CNF-PANI complexes. The authors demonstrated that the multiple features of the components resulted in a versatile material with high flexibility, strength, stretchability, and conductivity. They suggested that the ternary hybrid nanocomposites could be used as a sensor for human activity monitoring. Besides the electrically conductive capacity of electronic devices, an adequate heat dissipation in flexible electronic devices is crucial for practical applications. In this context, strong anchoring interactions of reduced graphene oxide (rGO) with Ag^+^ as well as the hydrogen bonding interaction between rGO and CNF was achieved by layer-by-layer (LBL) technique (Yang et al., 2020). According to this study, not only was the thermal conductive enhanced but also strength and flexibility. Chen et al. (2015) enhanced the conductivity and thermal stability properties in the final hybrid material. This was achieved upon incorporation of graphene into the conductive cellulose/polypyrrole composite. The obtained composite film was considered appropriate for electrodes due to its high specific capacitance and small impedance at 3.90 Ω. The authors suggested that the obtained flexible and lightweight nanocellulose composites could be employed as a supercapacitor component in a wide range of biomedical applications such as electronic skin (Hsu et al., 2019). Han L. et al. (2019) fabricated self-healable, biocompatible, strong and robust films made from CNC or CNF, PPy, and PVA for potential use as artificial electronic skin. The hydrogen bonds and Fe^3+^ chelation interactions and synergistic effects among the components enhanced the conductivity and mechanical property of the nanocomposite.

Hybrid nanocellulose/PANI/PEDOT core-shell nanorod film has been prepared by *in-situ* chemical oxidative polymerization. The fabricated nanofilm displayed multicolor changes at different applied potentials, transmittance modulation ability, short response time, and good cycling stability. According to the authors, the fabricated nanofilm has a broad range of applications mostly in smart windows and displays (Zhang S. et al., 2019).

## Nanocellulose-Based Luminescent Materials

During the recent decades, non-conjugated and conjugated polymers and nanoparticles with different chemical structures emitting colors in the full range of the visible region have been investigated in alternative processes for fabricating light-emitting materials. For the transition from traditional to foldable and flexible conductive and luminescent devices, new developments of bio-based sensors, organic solar cells, and organic field emission transistors are projected to occur rapidly (Liang et al., 2014; Thomas et al., 2018). Fluorescently functionalized nanocellulose has been developed to expand its feasibility for sophisticated applications such as flexible devices decorated with luminogens elements (Zhang et al., 2017; Li et al., 2019).

### Combination of Nanocellulose and Carbon Dots

**Figure 8:**
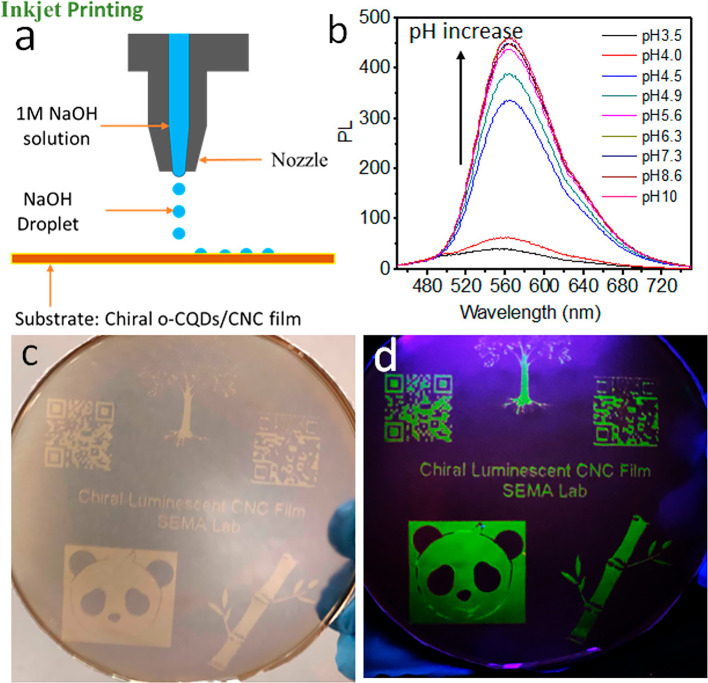
**(a)** Inkjet printing of luminescent CNC films. **(b)** Photoluminescent spectra of QD dispersion in different pH conditions. Visual appearance of luminescent CNC films under natural light **(c)** and UV light **(d)**. Reprinted with permission from Xiong et al. (2019). Copyright (2019) American Chemical Society.

Transparent, smooth, and fluorescent nanofilms have been fabricated from nanocellulose-carbon dots (NC-CD). Recently, CDs represent an emerging class of fluorescent materials with broad and strong UV absorbance (Feng et al., 2017). CDs are considered as a potential candidate to replace semiconductor quantum dots in several applications such as sensors, bioimaging, and drug delivery and optoelectronic devices (Jiang et al., 2016). It has been demonstrated that luminescent CDs can be covalently or physically attached to nanocellulose by different ways. The CDs modification not only provides active and anchor sites but also the prevent the agglomerations CDs within nanocellulose films. In 2016, for the first time, CDs were obtained using nanocellulose nanoparticles as the starting material (Jiang et al., 2016). Recently, CDs were obtained via hydrothermal method from pine residues and subsequently combined with PVA and CNF (Xu L. et al., 2020). The same method was used by Ng et al. (2020) to produce CDs and form a three-dimensional network along with MFC in PVA for building photoluminescent thin film as a tartrazine sensor. The importance of more research to demonstrate the potential of nanocellulose/quantum dot in biosensing applications has been pointed out (Junka et al., 2014). Heavy metal contamination grown as an environmental concern. In this context, Song et al. (2020) developed a CQDs/CNF composite for the adsorption and detection of Cr^3+^ in water. The authors investigated the synergic effect between adsorption and florescence to real-time monitor the presence of heavy metal. Carbon quantum dots have also been grafted onto oxidized cellulose nanofibrils for Fe^3+^ ion detection (Xue et al., 2020). The flexible and photoluminescent nanofilm was fabricated using a one-pot method without the need for any catalyst. Li and Hu (2019) and Yan et al. (2018) reported a type of multifunctional nanocellulose composite film decorated with CdTe (cadmium telluride) and CdS (cadmium sulfide), respectively. According to the authors, the highly photoluminescent, flexible and strong fabricated nanofilms could be employed for anti-counterfeit marking. Tetsuka (2015) obtained a highly luminescent flexible hybrid nanocomposite composed of amino-functionalized graphene quantum dots/CNF and clay for white-light emitting diodes. In a recent study, the physical interaction of positive charge on chitosan and negative charge on CDs was evaluated as potential green hydrogel for biomedical applications (Konwar et al., 2015). They found that chitosan–carbon dots films exhibited excellent properties such as UV–visible blocking, thermal stability and mechanical strength. According to a recent study, CNCs decorated covalently with ZnS quantum dots were embedded in PVA matrix to prepare nanocomposite films with antibacterial properties and bright blue fluorescence under the ultraviolet light (Xie et al., 2019). Recently, carbodiimide coupling was used to link CNCs and QDs to fabricate hybrid nanoparticles which can be employed in biological tracking and fluorescent material applications (Abitbol et al., 2017). Furthermore, it has been proposed that luminescent cellulose nanocrystals particles can partially replace carbon nanoparticles by reducing the final cost for the production in potential applications in a variety of fields such as biomedical, photosensors, and photocatalysis (da Silva Souza et al., 2018). Xiong et al. (2019) reported an emissive carbon quantum dots assembled with CNC nanostructure for fabricating flexible robust and strong chiral fluorescent materials. The films became highly fluorescent under alkaline conditions (Figure 8).

**Figure 9:**
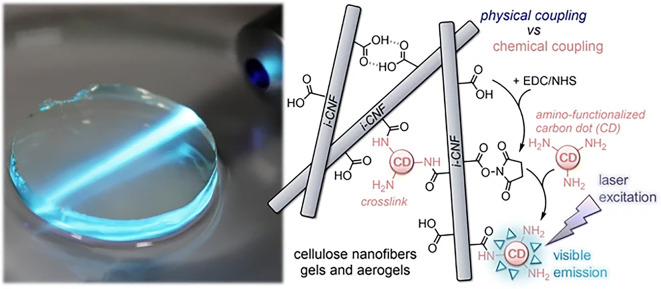
CNF aerogels with covalently coupled CDs under laser beam of 100 mW at 405 nm crossing through the soft gels, and scheme showing covalent bonds. Reprinted with permission from Quraishi et al. (2019). Copyright (2019) Springer Nature.

As shown is Figure 9, Quraishi et al. (2019) presented highly transparent bio-based hybrid films obtained by carboxylated CNF and decorated with amino-functional photoluminescent carbon dots. They concluded that the fabricated luminescent hybrid materials are promising candidates for sustainable volumetric display applications. This interesting and valuable technology can be suited to the development of holographic displays (Zhong et al., 2016).

### Combination of Nanocellulose and Luminescent Organic Polymers

Luminescent organic polymers have been reported as an alternative to CDs and metal particle for fluorescent devices (Li et al., 2019). Grigoray et al. (2017) have synthesized a light-responsive pulp fiber with light-controllable mechanical properties decorated with coumarin moieties. Fluorescent CNCs with carbazole and coumarin functionalities were prepared as an invisible security fiber for authenticity indicators that turn visible when being exposed to UV light (Sîrbu et al., 2016). Tong et al. (2020) fabricated a highly transparent hydrophobic cellulose film for application in flexible electroluminescent devices via free radical polymerization of vinyl-bearing cellulose. The authors suggested that the cellulose functionalized film has great potential applications in flexible transparent electronics. Recently, a fascinating technique was developed by incorporating dioxetanes as stress sensors into polymethyl acrylate/CNCs nanocomposite. Intriguingly, the nanocomposite emitted blue light when mechanical force was applied, and the emission intensity varied according to the tensile strain (Chen et al., 2020). The color variations of nanocomposites under different light and temperature conditions have also been explored for display applications. Recently, CNC particles were incorporated into ethylene vinyl alcohol copolymer (EVAL) and the resulting nanocomposite film was subsequently immersed in a solution of a mixture of photochromic/thermochromic powders and epoxy resins (Li et al., 2020). Nanocomposites of PVA and CNC were developed as sensor films which exhibited light emission intensity variations in response to changes in pH (Schyrr et al., 2014). The allyl functionalized nanocomposites could detect protease activity for applications in wound diagnosis. A simple and low-cost approach for the preparation of a coumarin-TOCNF /PVA membrane with effective fluorescence response was successfully achieved (Zhao et al., 2019). The robust and strong nanocomposite exhibited fluorescence under UV light with potential applications in wound diagnosis and other biomedical technologies. A ratiometric fluorescent nanopaper sensor was investigated and suggested as an artificial tongue for chemical discrimination applications (Abbasi-Moayed et al., 2018). Bacterial cellulose nanopaper as a substrate offered favorable conditions for developing artificial tongues because of its flexibility and transparency. CD grafted with Rhodamine B was incorporated into bacterial nanocellulose, and the resulting film exhibited extraordinary mechanical and physicochemical features for use as a fluorescent sensor for the detection of a variety of heavy metal ions (Abbasi-Moayed et al., 2018). Ionic liquid has also been employed as a green and reusable solvent for CNC film functionalization with amine groups via dip-and-pull process (Song et al., 2018). They developed a sensors that can change in color when exposed to formaldehyde and propanal (Song et al., 2018). Hoenders et al. (2018) revealed that tetrazole-functionalized CNF which underwent subsequent functionalization with maleimide-based compound led to self-reporting photo-induced phenomena, making it a promising application for photo-patterning of transparent nanofilm.

### Inorganic Hybrid Luminescent Nanocomposites

Nanocellulose-inorganic hybrid materials have been employed due to the multiple possible functions and synergistic effects among their components. A transparent nanofilm was prepared from TOCNF films with sodium carboxylate groups by immersing the films in nitrate complexes of europium solution (Yang et al., 2018). The strong ionic interactions between the carboxylate groups and Eu^3+^ ions improved the mechanical properties of the nanofilms. In addition, the exceptional photoluminescence, moisture resistance and thermal stability offered new and exciting opportunities for realizing multifunctional films in electric devices (Yang et al., 2018). Functionalized TOCNF film with stable fluorescence properties was fabricated by *in-situ* synthesis of a Eu-based metal–organic framework (Wang et al., 2020b). The nanofilm exhibited high selectivity for copper ion detection over a wide range of interfering metal ions. Surface modified SrAl_2_O_4_: Eu^2+^, Dy^3+^ phosphors using (3-aminopropyl) trimethoxy-silane (APTMS) were incorporated into the TEMPO-oxidized cellulose nanofibril (TOCNF) matrix using a green sol-gel method to fabricate strong and flexible films with long-lasting afterglow luminescence. According to the authors, the resulting flexible, thermal responsive, and light- induced nanocellulose films may open up new possibilities for temperature sensor devices in harsh industrial applications (Zhang L. et al., 2019). In another study, ZnO/TONC composite films showed photoluminescence when excited by UV light (Ning et al., 2018). Fedorov et al. (2019) prepared polymer-inorganic films based on CNC and CNF and strontium fluoride (SrF_2_): holmium (Ho) powders. According to the authors, the resulting films can be employed for visualization of laser radiation. Zhang et al. (2017) developed transparent and flexible nanofilms from lanthanide complex grafted CNF with ability to absorb and convert UV light into pure red-light emission. Interestingly, the coefficient of thermal expansion (6.39 ppm K^−1^) of the resulting film was lower than that of films fabricated from fossil-based materials for red light-emitting devices.

## Perspectives and Conclusions

Fascinating biopolymers and sustainable raw materials have been extensively studied in response to a global need for low cost and renewable material as a feedstock for next generation materials. The reinforcing effect of nanocellulose has been frequently employed in less sophisticated products and materials for food applications such as packaging. The energy consumed during nanocellulose fabrication may cause it to be commercially impracticable in its utilization for certain applications. Therefore, it is reasonable to assume that the use of nanocellulose for more complex and sophisticated applications can compensate its actual price and not impose any constraints in the production of high-value products on a large-scale. In this context, nanocellulose, in the form of nanocrystal or nanofiber, has emerged as a promising material and platform for the next generation of high-performing and energy-related devices. Functionalized nanocellulose can be employed as an alternative to expensive conventional petroleum-based electronic components and devices. Commercialization of the nanocellulose-based advanced materials has been suggested as the next big breakthrough. It is well-known that CNF exhibits outstanding mechanical robustness, flexibility and provides load-bearing ability. In this sense, CNF has been employed to fabricate functional free-standing films, combining multiple features by anchoring nanoparticles either by blending or grafting. On the other hand, the percolation ability of CNC as well as its tunable surface chemistry makes it particularly interesting for the development of functional materials for green and sustainable chemistry, electronics, medicine and food, among others. Having said that, the tradeoff between electrical conductivity and optical transmittance is still a major challenge in exploiting the full potential of nanocellulose-based conductive films. In this context, it is expected that further studies will be conducted to fully integrate nanocellulose-based materials into society and commerce. In terms of luminescent nanocellulose films, the current scenario reveals that substantial effort has been invested in the optical sensing and detection applications. However, it is worth mentioning that a number of studies have focused on more sophisticated applications such as screen-printing, mechano-luminescent sensors, and holographic displays. In addition, it was found that TEMPO-mediated oxidation of nanocellulose was extensively used for the development of nanocellulose-based luminescent materials. The current challenge is building high-performance materials almost exclusively constructed from renewable materials. To build flexible and robust devices, multiple components with specific features need to be integrated into a single device. In this context, the combination of features such as conductivity and luminescence is crucial for the next-generation green electronics. In addition, current studies reveal a trend toward renewable materials as starting materials for CDs, CNT, and graphene production. This is particularly important to reduce the carbon footprint without affecting hybrid nanocomposite properties. In this review, a variety of promising and fascinating approaches have been demonstrated. Development of novel bio-based materials demand different features and performance from cellulose based elements. Since it is not feasible fabricate high-performance bio-based devices exclusively made of nanocellulose, the use of nanocellulose as template to control the surface and anchor functional materials can enhance the performance, add multiple functionalities and consequently expand the uses of nanocellulose-based films while meeting environmental requirement. Herein, we demonstrated that functional nanocellulose will open new opportunities for the design of flexible energy and electronic materials, which is very important in developing the future generation of green materials.

## Author Contributions

OD took the lead in writing the manuscript. SK, AL, WY, JT, and MS provided critical feedback and helped shape the research, analysis, and manuscript.

## Conflict of Interest

The authors declare that the research was conducted in the absence of any commercial or financial relationships that could be construed as a potential conflict of interest.
